# Supplementary material for: Genomic Analysis of 18th-Century Kazakh Individuals and Their Oral Microbiome
Source: Biology (Basel). 2021 Dec 14;10(12):1324. doi: 10.3390/biology10121324 (PMC8698332; doi:10.3390/biology10121324)
Supplement: Supplementary file 1 [file biology-10-01324-s001.zip › biology-1510291-supplementary.pdf]

Supplementary information

Genomic analysis of 18th-century Kazakh individuals and their oral microbiome

Anna White, Toni de-Dios, Pablo Carrión, Gian Luca Bonora, Laia Llovera, Elisabetta Cilli, Esther Lizano, Maral K. Khabdulina, Daniyar T. Tleugabulov, Iñigo Olalde, Tomàs Marquès-Bonet, François Balloux, Davide Pettener, Lucy van Dorp, Donata Luiselli, Carles Lalueza-Fox

Table S1. Available C14 dates of Kuygenzhar individuals; datings were done at the Centro di Datazione e Diagnostica (CEDAD) de l'Università del Salento.

**Sample name**	**Radiocarbon Age (BP)***	**Year (CE)**	**Lab code**	**δ13C (‰)**
**B2_B3**	237 ± 40	1713 ± 40	LTL20392A	-19.8 ± 0.5
**B2_B5**	166 ± 40	1784 ± 40	LTL20393A	-21.7 ± 0.5
**B2_B6**	165 ± 40	1785 ± 40	LTL20394A	-17.8 ± 0.5

*1950 as present time

Table S2. Genetic sex determination of the five Kuygenzhar individuals.

**Individual**	**Nseqs**	**NchrY+NchrX**	**NchrY**	**R_y**	**SE**	**95% CI**	**Assignment**
**B2_B1**	2346622	65198	6022	0.0924	0.0011	0.0901-0.0946	XY
**B2_B3**	11448368	575423	1530	0.0027	0.0001	0.0025-0.0028	XX
**B2_B5**	13838347	382064	35188	0.0921	0.0005	0.0912-0.093	XY
**B2_B6**	24913319	689321	63824	0.0926	0.0003	0.0919-0.0933	XY
**B2_B7**	23370803	640118	59474	0.0929	0.0004	0.0922-0.0936	XY

Table S3. Estimates of contamination in the Kuygenzhar individuals.

**X Chromosome Heterozygosity**			**Mitochondrial Contamination**			
**Sample**	Contamination Estimate	Standard deviation	Average Contamination	Lower Boundary	Upper Boundary	
**B2_B1**	No data	No data	No data	No data	No data	*
**B2_B3**	NA (Female)	NA (Female)	0	0	95.00%	*
**B2_B5**	0.011171	5.789074e-03	0	0	1.50%	*
**B2_B6**	0.002998	0.00540908	26.50%	20.50%	32.50%	
**B2_B7**	0.004636	0.00676443	0	0	95.00%	*

*With an insufficient amount of data contamination cannot be estimated or the range of possible contamination may be wide.

Table S4. Mitochondrial and Y-Chromosome haplogroups assigned to the Kuygenzhar individuals.

Individual	Mitochondrial Haplotype	Y-Chromosome Haplotype
**B2_B3**	G2a2	Female
**B2_B5**	R2+13500	G1b-L830
**B2_B6**	U5a1a1h	C2a1a3-M504
**B2_B7**	T2b28	C2

Table S5. Two-way ancestry model for the Kuygenzhar individuals with qpAdm. Outgroups: Mbuti.DG, ONG.SG, Ami.DG, Mixe.DG, Israel_Natufian_published, Iran_GanjDareh_N, Turkey_N, Italy_North_Villabruna_HG.

Target	Ref1	Ref2	p-value	Coeff1	Coeff2
B2_B3	Kyrgyzstan_Medieval_Nomad.SG	Kazakhstan_His.SG	0.4839	0.283 ± 0.027	0.717 ± 0.027
B2_B5	Kyrgyzstan_Medieval_Nomad.SG	Kazakhstan_His.SG	0.0658	0.286 ± 0.024	0.714 ± 0.024
B2_B6	Kyrgyzstan_Medieval_Nomad.SG	Kazakhstan_His.SG	0.1820	0.331 ± 0.042	0.558 ± 0.042
B2_B7	Kyrgyzstan_Medieval_Nomad.SG	Kazakhstan_His.SG	0.2096	0.283 ± 0.047	0.717 ± 0.047
Kuygenzhar	Kyrgyzstan_Medieval_Nomad.SG	Kazakhstan_His.SG	0.6116	0.408 ± 0.020	0.592 ± 0.020

Table S6. Coverage for the three Red Complex bacteria (*Tannerella forsythia*, *Treponema denticola* and *Porphyromonas gingivalis*) in each individual sample.

	Tannerella forsythia		
	**Average Depth**	**Reference Covered**	**Q30 Reads**
B2-B1	0,0032	0.31%	132
B2-B3	11,4159	66.29%	391726
B2-B5	0,0806	7.23%	3366
B2-B6	0,3282	23.18%	12304
B2-B7	8,8889	83.23%	318145

	Treponema denticola		
	**Average Depth**	**Reference Covered**	**Q30 Reads**
B2-B1	0,0011	0.11%	37
B2-B3	3,9331	84.68%	110442
B2-B5	0,0236	2.31%	689
B2-B6	0,1726	15.06%	5240
B2-B7	0,9959	49.94%	29052

	Porphyromonas gingivalis		
	**Average Depth**	**Reference Covered**	**Q30 Reads**
B2-B1	0,0026	0.25%	74
B2-B3	4,3217	82.07%	116330
B2-B5	0,0112	1.04%	352
B2-B6	0,0834	7.76%	2663
B2-B7	0,8203	32.76%	22689

Table S7. List of all *Tannerella forsythia* samples used in the study. Origin of the sample is specified by their publication or their assembly identifier.

Sample	Approximate Dating	Country	Specie	Average Depth	Dataset	Article / Assembly ID
**ECO002**	-7500	Spain	H. sapiens	1,4058X	Low Quality	1
**ECO004**	-7820	Spain	H. sapiens	0,532X	Low Quality	1
**ESA008**	1700	Morocco	H. sapiens	0,5769X	Low Quality	1
**GOY005**	-41125	Belgium	H. neanderthal	1,6819X	Low Quality	1
**LIS001**	1700	Portugal	H. sapiens	1,3259X	Low Quality	1
**LIS003**	1700	Portugal	H. sapiens	2,1888X	Low Quality	1
**MOA001**	-2471	Ethiopia	H. sapiens	0,7418X	Low Quality	1
**OAK003**	-4208		H. sapiens	1,2039X	Low Quality	1
**OAK005**	-4600		H. sapiens	3,2383X	Low Quality	1
**OFN001**	-6291	Germany	H. sapiens	0,7768X	Low Quality	1
**PYK001**	1800	South	H. sapiens	0,6557X	Low Quality	1
**PYK002**	1800		H. sapiens	4,9867X	High Quality	1
**PYK003**	1800		H. sapiens	3,155X	Low Quality	1
**PYK005**	1800		H. sapiens	1,3959X	Low Quality	1
**TAF008**	-12000	Morocco	H. sapiens	1,9884X	Low Quality	1
**VLC001**	2016	Spain	H. sapiens	29,8478X	High Quality	1
**VLC002**	2016	Spain	H. sapiens	1,1704X	Low Quality	1
**VLC004**	2016	Spain	H. sapiens	2,1251X	Low Quality	1
**VLC005**	2016	Spain	H. sapiens	19,3391X	High Quality	1
**VLC008**	2016	Spain	H. sapiens	13,7029X	High Quality	1
**VLC009**	2016	Spain	H. sapiens	5,9612X	High	1

					Quality	
**B61**	1050	Germany	H. sapiens	4,1792X	High Quality	2
**G12**	1050	Germany	H. sapiens	4,0269X	High Quality	2
**CS21**	1812	England	H. sapiens	5,2219X	High Quality	2
**CS40**	1812	England	H. sapiens	6,5113X	High Quality	2
**B2_B3**	1713	Kazakhstan	H. sapiens	11,4159X	High Quality	This study
**B2_B6**	1785	Kazakhstan	H. sapiens	0,3282X	Low Quality	This study
**B2_B7**	1750	Kazakhstan	H. sapiens	8,8889X	High Quality	This study
**CA13**	-565	Mexico	H. sapiens	0,2039X	High Quality	3
**TO-2417Q**	1250	Mexico	H. sapiens	14,985X	High Quality	3
**TO-2417J**	-900	Mexico	H. sapiens	8,4118X	High Quality	3
**TO333O**	-700	Mexico	H. sapiens	4,3302X	Low Quality	3
**TLA01**	1487	Mexico	H. sapiens	3,378X	Low Quality	3
**TLA22**	1455	Mexico	H. sapiens	2,7067X	Low Quality	3
**HSJN194**	1548	Mexico	H. sapiens	0,84X	Low Quality	3
**HSJN240**	1525	Mexico	H. sapiens	2,2505X	Low Quality	3
**CO09**	1800	Mexico	H. sapiens	0,1489X	High Quality	3
**CO20**	1800	Mexico	H. sapiens	2,5809X	High Quality	3
**3313**	~ 2010	Japan	H. sapiens	Assembly	High Quality	ASM154787v1
**UB20**	2013	USA	H. sapiens	Assembly	High Quality	TFUB20
**KS16**	~ 2010	Japan	H. sapiens	Assembly	High Quality	ASM154785v1
**OH2617_COT-023**	2012	England	Canis lupus	Assembly	High Quality	ASM386002v1
**UB22**	2013	USA	H. sapiens	Assembly	High Quality	TFUB22
**UB4**	2013	USA	H. sapiens	Assembly	High Quality	TFUB4
**WW10960**	~ 2010	England	H. sapiens	Assembly	High Quality	ASM252929v1
**WW11663**	~ 2010	England	H. sapiens	Assembly	High Quality	ASM252908v1

**92A2**	1986	USA	H. sapiens	Assembly	High Quality	ASM23821v1
**9610**	1990	USA	H. sapiens	Assembly	High Quality	ASM193878v1
**VE42**	150	Mexico	H. sapiens	1,0167X	Low Quality	3
**UAQG**	NA	Mexico	H. sapiens	1,88X	Low Quality	3

Article	ID
**J. A. F. Yates, et al., The evolution and changing ecology of the African hominid oral microbiome. Proc. Natl. Acad. Sci. U. S. A. 118, e2021655118 (2021).**	1
**C. Warinner, et al., Pathogens and host immunity in the ancient human oral cavity. Nat. Genet. 46, 336–344 (2014).**	2
**M. Bravo-Lopez, et al., Paleogenomic insights into the red complex bacteria Tannerella forsythia in Pre-Hispanic and Colonial individuals from Mexico. Philos. Trans. R. Soc. B Biol. Sci. 375, 20190580 (2020).**	3

Table S8. List of recombinant tracks detected using ClonalFrameML, with their starting and finishing position.

Start	End		Start	End		Start	End
495	42003		1046209	1046347		2042157	2054741
42690	45502		1048396	1050841		2055424	2076427
46141	64412		1053170	1053473		2085896	2086222
100903	100953		1058472	1059515		2103969	2133181
114237	115255		1060516	1086407		2140569	2151453
135232	139335		1088210	1106270		2152592	2160338
142200	142257		1109372	1123511		2162869	2197942
145176	145210		1124705	1138188		2199205	2203617
148132	153018		1141413	1142987		2213970	2215754
153048	154075		1145491	1146206		2217182	2232911
156187	156979		1165926	1184285		2245609	2255734
158160	166016		1185267	1187297		2261568	2291274
169282	183681		1187930	1190060		2292789	2306979
187493	190897		1190634	1191098		2309228	2318838
192877	226071		1191287	1196328		2321900	2322779
238155	260883		1199660	1201687		2323325	2323671
263953	266293		1203411	1210691		2326953	2333591
268327	273990		1212090	1233925		2335333	2338584
275203	294419		1242769	1252063		2340784	2345121
294986	295030		1254613	1261003		2345595	2350084
297503	298611		1272489	1293981		2354121	2357406
300217	300359		1301645	1312692		2362546	2386686
302694	304369		1313783	1314593		2391257	2394264
304959	305255		1364708	1365255		2404420	2423556
307827	310347		1365651	1369744		2424618	2440301
311523	348875		1369970	1370636		2440750	2461033
351850	357945		1371677	1371815		2475000	2492353
360472	361291		1374127	1380070		2498530	2498759
362859	363922		1380278	1380340		2500093	2500309
379253	379289		1382103	1423167		2506640	2507786
393485	395928		1425471	1426725		2513438	2536624
396704	403118		1429045	1447263		2537281	2543437
404488	415604		1450559	1450787		2544042	2544174
416314	444178		1453221	1454571		2552290	2561852
445571	466803		1454848	1469482		2563765	2585038
471230	485410		1471326	1476997		2586720	2591367
487469	500386		1478868	1493901		2597332	2611702
502339	535295		1507059	1507322		2617236	2619851
536533	539293		1517561	1518761		2628837	2652861
540631	546741		1519394	1521821		2653755	2655195

548650	548767		1529704	1559344		2657130	2679712
**Start**	**End**		**Start**	**End**		**Start**	**End**
550909	561035		1566285	1568910		2681269	2691756
562379	566808		1572051	1577747		2697159	2698196
573503	590840		1580285	1581435		2698336	2698441
591658	608563		1587660	1598007		2703106	2703616
610006	611963		1599090	1604157		2703911	2704389
614990	616577		1605306	1625158		2710977	2711227
619131	621962		1626046	1641802		2714677	2735206
623084	624652		1643873	1646372		2735317	2735479
627380	627508		1648289	1674909		2739084	2739340
629966	642231		1676010	1678624		2739536	2739764
649693	653296		1682441	1683494		2741623	2761322
656857	664322		1685725	1700222		2764342	2795801
692086	697707		1700279	1714258		2797886	2823519
698902	731250		1718956	1721516		2825382	2828786
732219	733113		1722496	1753517		2829847	2831930
742740	743134		1755728	1755749		2833057	2835106
750332	751393		1756612	1756984		2836227	2841173
757535	771040		1757404	1757674		2842993	2852152
771902	772029		1758777	1764076		2853747	2854302
772176	773039		1765766	1806002		2855765	2880567
773393	774178		1806388	1806890		2896469	2896495
775359	784835		1807419	1809878		2896529	2899218
785021	786248		1818634	1819218		2900627	2993497
789155	791808		1820523	1820586		2994603	3001606
794166	794794		1822643	1824295		3002769	3006671
796588	803854		1826331	1827255		3011082	3041429
810686	821600		1827574	1850059		3043523	3058751
822665	829244		1851071	1860715		3060350	3094131
832767	840408		1862458	1862617		3095993	3110475
843902	848701		1864391	1864568		3118616	3140429
850554	851372		1866399	1871097		3141795	3141917
852914	861170		1874337	1884027		3144933	3170919
863100	863820		1887513	1913425		3174803	3207929
864617	896228		1913693	1913765		3209014	3215048
899239	911309		1914208	1929115		3218622	3224792
917076	918085		1930750	1938771		3227639	3231348
921325	940699		1940682	1964710		3238676	3242254
960293	967372		1968094	1968702		3249201	3253628
970584	991072		1970913	1971591		3260687	3262342
993255	994323		1987063	1995077		3267531	3268743
994326	996698		1996249	2004724		3278240	3279096
997349	1024774		2008590	2014252		3313545	3330813
1026165	1026391		2015788	2028584		3333016	3342298

1028476	1032313		2029357	2031575		3344574	3357610
**Start**	**End**		**Start**	**End**		**Start**	**End**
1033462	1038518		2033202	2034080		3363873	3373352
1039629	1044802		2034405	2039790		3374472	3405521

Table S9. Dating of the *T. forsythia* phylogeny nodes estimated using BactDating, including the upper and lower confidence interval boundaries (± 95%).

Node	Date	Lower 95% CI	Upper 95% CI	Node	Date	Lower 95% CI	Upper 95% CI
**[1,]**	1800	1800	1800	**[24,]**	-11209,9	-20219,4	-2200,47
**[2,]**	2010	2010	2010	**[25,]**	-3585,56	-6121,01	-1050,11
**[3,]**	2010	2010	2010	**[26,]**	-2149,65	-3387,01	-912,289
**[4,]**	1990	1990	1990	**[27,]**	-620,43	-1802,55	561,6907
**[5,]**	2016	2016	2016	**[28,]**	-198,473	-1176,13	779,1804
**[6,]**	2016	2016	2016	**[29,]**	125,402	-640,669	891,4741
**[7,]**	2013	2013	2013	**[30,]**	597,538	180,6718	1014,405
**[8,]**	2013	2013	2013	**[31,]**	786,88	526,9154	1046,844
**[9,]**	2016	2016	2016	**[32,]**	1178,22	795,0726	1561,361
**[10,]**	2010	2010	2010	**[33,]**	1321,83	952,3872	1691,273
**[11,]**	2010	2010	2010	**[34,]**	1410,33	1072,437	1748,225
**[12,]**	2013	2013	2013	**[35,]**	1510,93	1239,833	1782,02
**[13,]**	1812	1812	1812	**[36,]**	1582,54	1338,029	1827,059
**[14,]**	2010	2010	2010	**[37,]**	1685,38	1480,073	1890,681
**[15,]**	1812	1812	1812	**[38,]**	1789,23	1620,357	1958,108
**[16,]**	1050	1050	1050	**[39,]**	1892,2	1777,266	2007,137
**[17,]**	2016	2016	2016	**[40,]**	1855,74	1724,251	1987,235
**[18,]**	1750	1750	1750	**[41,]**	1843,49	1678,145	2008,837
**[19,]**	1713	1713	1713	**[42,]**	1701,63	1471,611	1931,645
**[20,]**	1050	1050	1050	**[43,]**	1800,69	1616,055	1985,326
**[21,]**	-900	-900	-900	**[44,]**	1900,82	1793,2	2008,436
**[22,]**	1250	1250	1250	**[45,]**	1847,48	1688,939	2006,021
**[23,]**	2010	2010	2010				

Table S10. All genes of *Tannerella forsythia* used to determine the presence or absence of virulence, antibiotic resistance and glycosylation genes.

**Annotation 92A2**	**Gene name**	**Gene function**	**Gene description**
**BFO_RS07215**	tfsB	Virulence gene	S-layer glycoprotein subunit TfsB
**BFO_RS15610**	tfsA	Virulence gene	S-layer glycoprotein subunit TfsA
**BFO_2272**	susB	Virulence gene	alpha-glucosidase
**BFO_1210**	siaHI	Virulence gene	oxidoreductase
**AB001892.1**	prtH	Virulence gene	
**BFO_2207**	nanH	Virulence gene	sialidase
**BFO_RS15140**	miropsin-2	Virulence gene	
**BFO_RS14385**	miropsin-1	Virulence gene	
**BFO_RS15130**	mirolysin	Virulence gene	
**BFO_RS15675**	mirolase	Virulence gene	
**BFO_RS07535**	methylglyoxal	Virulence gene	
	karilysin	Virulence gene	
**BFO_1923**	hexA	Virulence gene	beta-hexosaminidase
**BFO_1437**	groEL	Virulence gene	60 kDa chaperone family
**BFO_2737**	fucO	Virulence gene	alpha-L-fucosidase
**BFO_RS05090**	forsilysin	Virulence gene	
**BFO_2068**	enolase	Virulence gene	2-phospho-D-glycerate in glycolysis
	bspA_2	Virulence gene	
**BFO_RS14480**	bspA	Virulence gene	leucine-rich-repeat family virulence factor BspA
**BFO_2182**	bglX	Virulence gene	beta-D-glucoside glucohydrolase
**BFO_2553**	beta-Gal_3	Virulence gene	beta-galactosidase
**BFO_0450**	beta-Gal_2	Virulence gene	beta-galactosidase
**BFO_1381**	beta-Gal_1	Virulence gene	glycoside hydrolase family 2
**BFO_2947**	alpha-Man_2	Virulence gene	alpha-mannosidase
**BFO_0315**	alpha-Man_1	Virulence gene	alpha-mannosidase
**BFO_3112**	abfA	Virulence gene	
**BFO_1930**	TF2930	Virulence gene	glutamyl-tRNA synthetase
**BFO_1924**	TF2926	Virulence gene	serine hydroxymethyltransferase
**BFO_1388**	TF2392	Virulence gene	alpha-galactosidase
**BFO_1007**	TF2014	Virulence gene	peptidase S9

**BFO_0711**	TF1723	Virulence gene	alpha-rhamnosidase
**BFO_0705**	TF1717	Virulence gene	DNA mismatch repair protein MutS
**BFO_1055**	TF1705	Virulence gene	transcriptional regulator
**BFO_0574**	TF1589	Virulence gene	Tanf_05795
**BFO_0490**	TF1502	Virulence gene	glycoside hydrolase
**BFO_0418**	TF1439	Virulence gene	
**BFO_0395**	TF1416	Virulence gene	starch-binding protein
**BFO_0300**	TF1320	Virulence gene	glycoside hydrolase
**BFO_3356**	TF1012	Virulence gene	lysyl-tRNA synthetase
**BFO_3298**	TF0960	Virulence gene	beta-galactosidase
**BFO_3080**	TF0751	Virulence gene	peptidase S9
**BFO_2968**	TF0636	Virulence gene	beta-galactosidas
**BFO_2686**	TF0371	Virulence gene	beta-glycosidase
**BFO_2183**	TF0015	Virulence gene	membrane protein
**BFO_0996**	folA	Antbiotic resistance	Dihydrofolate reductase
**BFO_1541**	rpsL	Antbiotic resistance	SSU ribosomal protein S12p
**BFO_1543**	fusA	Antbiotic resistance	Translation elongation factor G
**BFO_1544**	rpsJ	Antbiotic resistance	SSU ribosomal protein S10p
**BFO_2029**	dxr	Antbiotic resistance	1-deoxy-D-xylulose 5-phosphate reductoisomerase
**BFO_2698**	rho	Antbiotic resistance	Transcription termination factor Rho
**BFO_2869**	rmsG	Antbiotic resistance	16S rRNA (guanine(527)-N(7))-methyltransferase
**BFO_1132**		Antbiotic resistance	3-oxoacyl-[acyl-carrier-protein] synthase
**BFO_1235**	tetQ	Antbiotic resistance	Tetracycline resistance, ribosomal protection type
**BFO_1091**	fabF	Antibiotic resistance	3-oxoacyl-[acyl-carrier-protein] synthase, KASII
**BFO_1348**	folP	Antibiotic resistance	Dihydropteroate synthase
**BFO_1939**	tuf	Antibiotic resistance	Translation elongation factor Tu
**BFO_1947**	rpoB	Antibiotic resistance	DNA-directed RNA polymerase beta subunit
**BFO_1948**	rpOC	Antibiotic resistance	DNA-directed RNA polymerase beta' subunit
**BFO_2280**	OxyR	Antibiotic resistance	Hydrogen peroxide-inducible genes activator
**BFO_3290**		Antibiotic resistance	D-alanine--D-alanine ligase

**BFO_0550**		Antibiotic resistance	Glycerophosphoryl diester phosphodiesterase
**BFO_1314**		Antibiotic resistance	3-oxoacyl-[acyl-carrier-protein] synthase, KASII
**BFO_1782**		Antibiotic resistance	Enoyl-[acyl-carrier-protein] reductase [NADH]
**BFO_2690**		Antibiotic resistance	Glycerophosphoryl diester phosphodiesterase
**BFO_1561**	rplF	Antibiotic resistance and essential gene	LSU ribosomal protein
**BFO_1695**	gyrB	Antibiotic resistance and essential gene	DNA gyrase subunit B
**BFO_2872**	gyrA	Antibiotic resistance and essential gene	DNA gyrase subunit A
**BFO_2936**	ileS	Antibiotic resistance and essential gene	Isoleucyl-tRNA synthetase
**BFO_RS00485**		Glycosylation gene	glycosyltransferase group 1 family protein
**BFO_RS00535**		Glycosylation gene	hypothetical protein
**BFO_RS00550**		Glycosylation gene	glycosyl transferase
**BFO_RS02100**		Glycosylation gene	hypothetical protein
**BFO_RS02105**		Glycosylation gene	penicillin-binding protein 1C
**BFO_RS02135**		Glycosylation gene	mannosyltransferase
**BFO_RS02420**		Glycosylation gene	glycosyl transferase family 1
**BFO_RS02430**		Glycosylation gene	glycosyl transferase family 1
**BFO_RS02435**		Glycosylation gene	hypothetical protein
**BFO_RS08625**		Glycosylation gene	hypothetical protein
**BFO_RS08630**		Glycosylation gene	glycosyl transferease family 2
**BFO_RS14090**		Glycosylation gene	hypothetical protein
**BFO_RS08670**		Glycosylation gene	hypothetical protein
**BFO_RS08675**		Glycosylation gene	glycosyltransferase group 1 family protein
**BFO_RS08680**		Glycosylation gene	glycosyl transferase
**BFO_RS10550**		Glycosylation gene	hypothetical protein
**BFO_RS10555**		Glycosylation gene	UDP-N-acetylglucosamine 2-epimerase (non-hydrolyzing)
**BFO_RS10600**		Glycosylation gene	glycosyl transferase

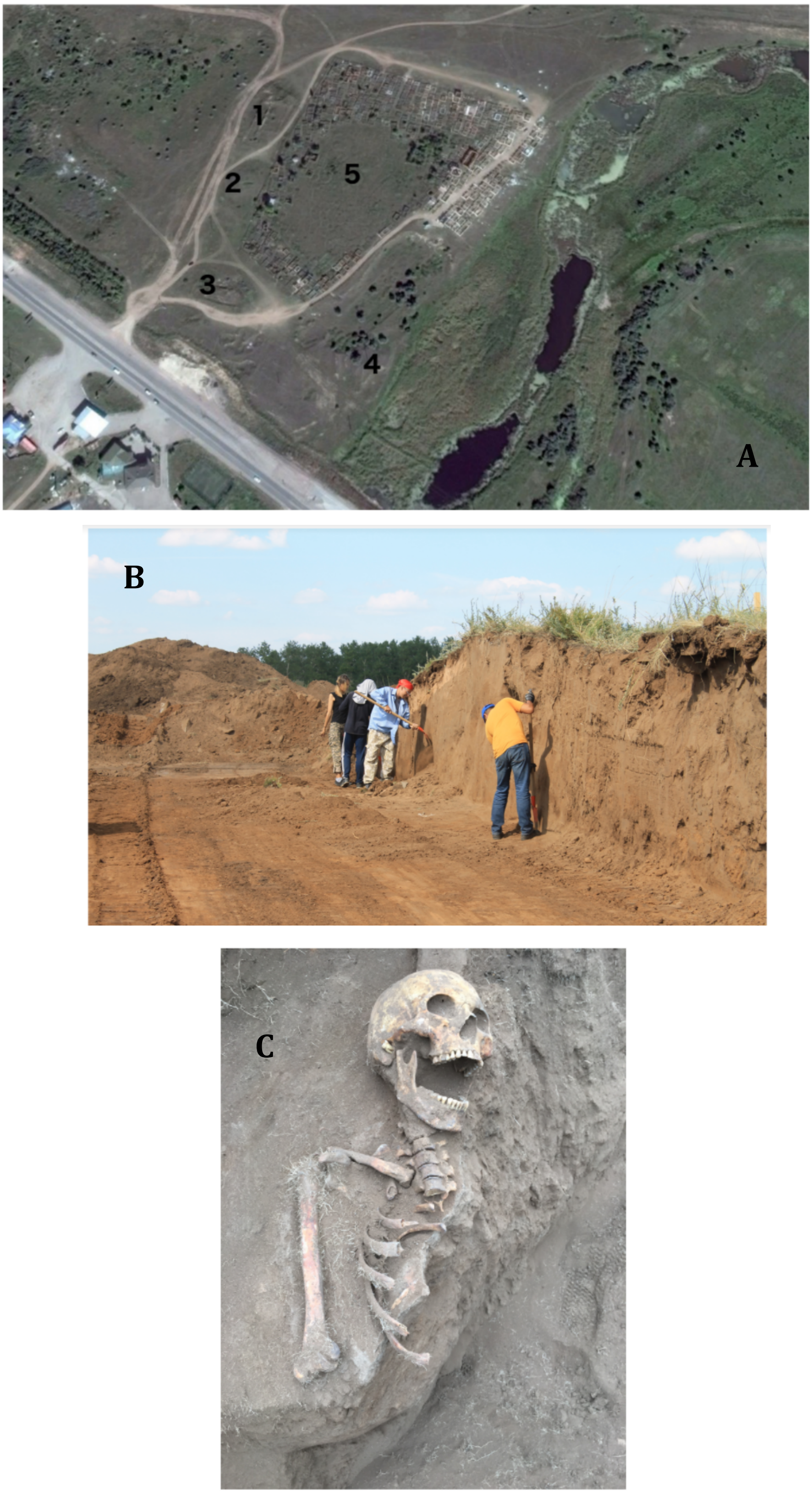

Figure S1. Excavation of the five individuals in Kuygenzhar, Kazakhstan. **A.** Aerial photo of the burial grounds, the kurgan excavated is located at 2. **B.** Excavation of the kurgan number 2 (1,3,4 and 5 numbers correspond to other, different barrows). **C.** One of the five individuals that was excavated.

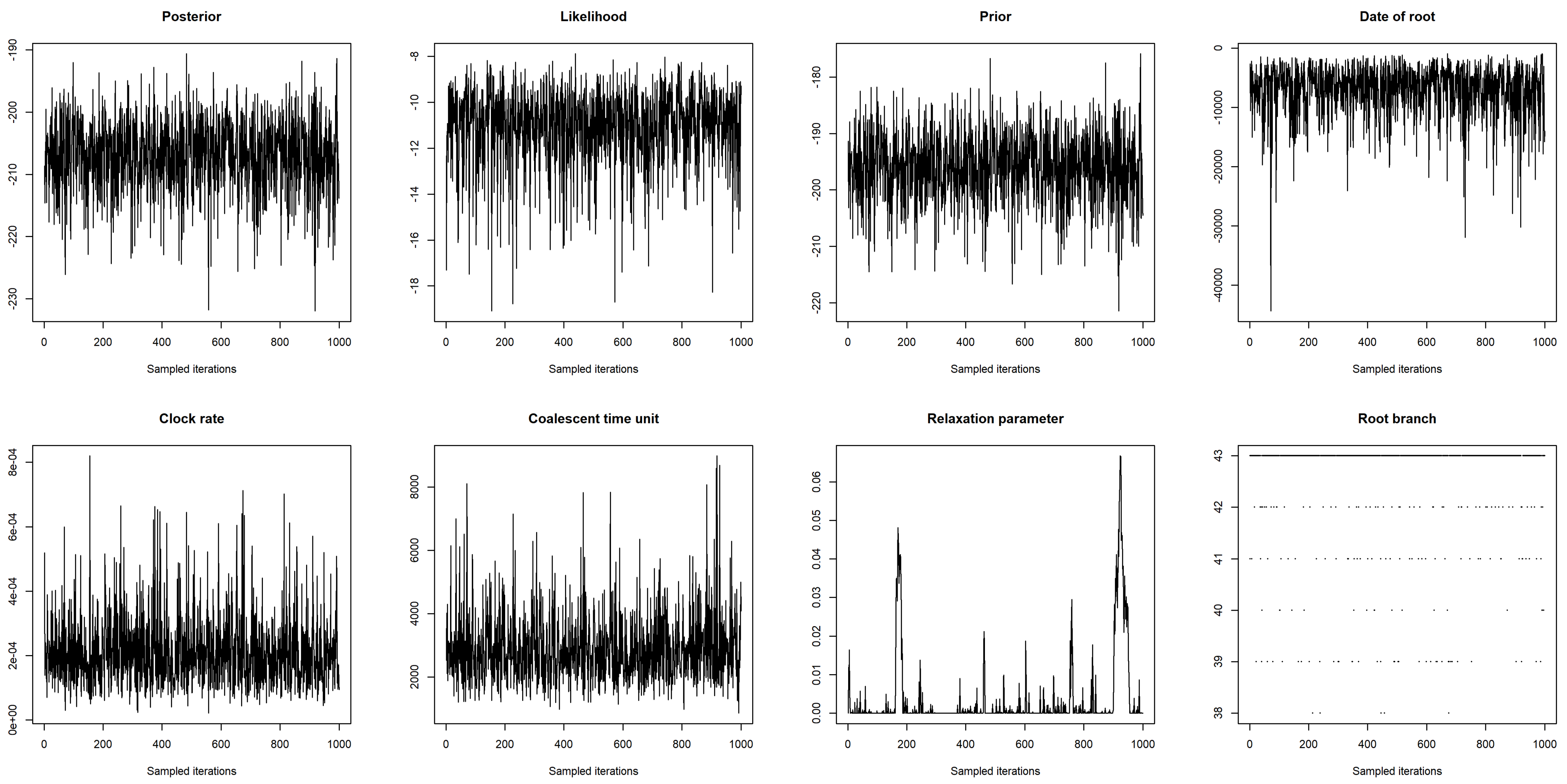

Figure S.2. MCMC Parameters convergence after 10.000,000 iterations

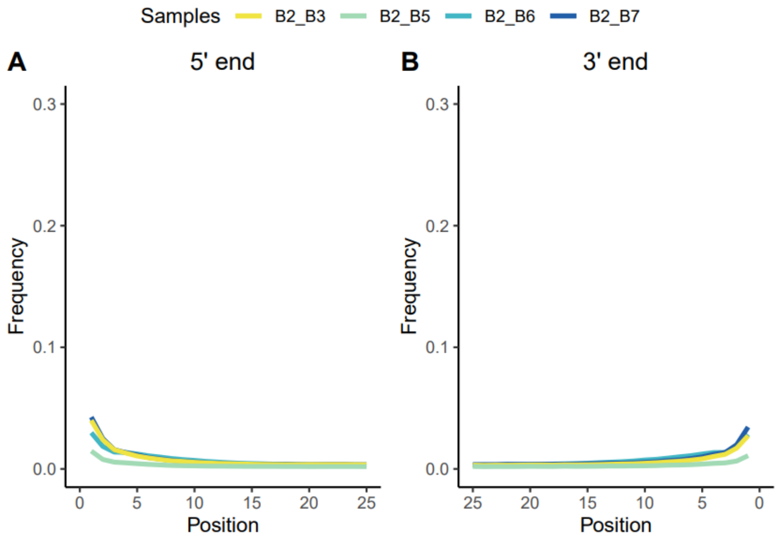

Figure S3. Post-mortem damage at the end of the DNA sequences. The frequency of substitutions C to T, at the 5' end (**A**), and G to A, at the 3' end (**B**). The substitutions at the end of DNA molecules show a damage pattern consistent with ancient DNA.

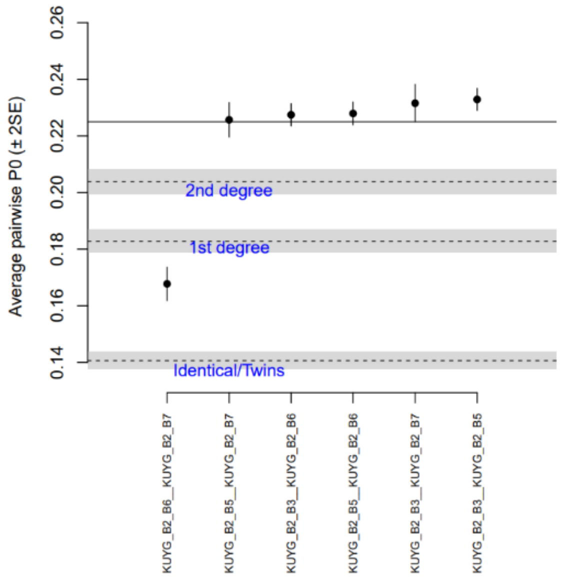

Figure S4. Kinship analysis between B2_B3, B2_B5, B_6 and B2_B7 identifies a first-degree relationship between B2_B6 and B2_B7. Since they are both male and have a different mitochondrial haplogroup they can be identified as father and son.

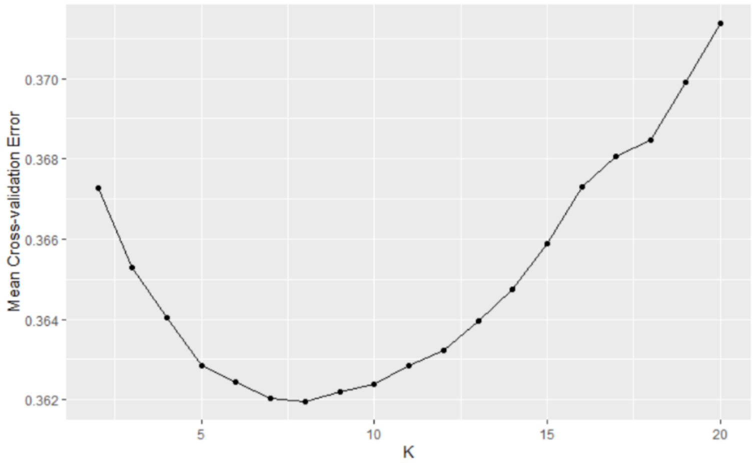

Figure S5. Mean cross-validation error to determine the most likely value of K for the ADMIXTURE analysis. These mean cross-validation errors indicate K=7 or K=8 as the best supported value for K of those tested.

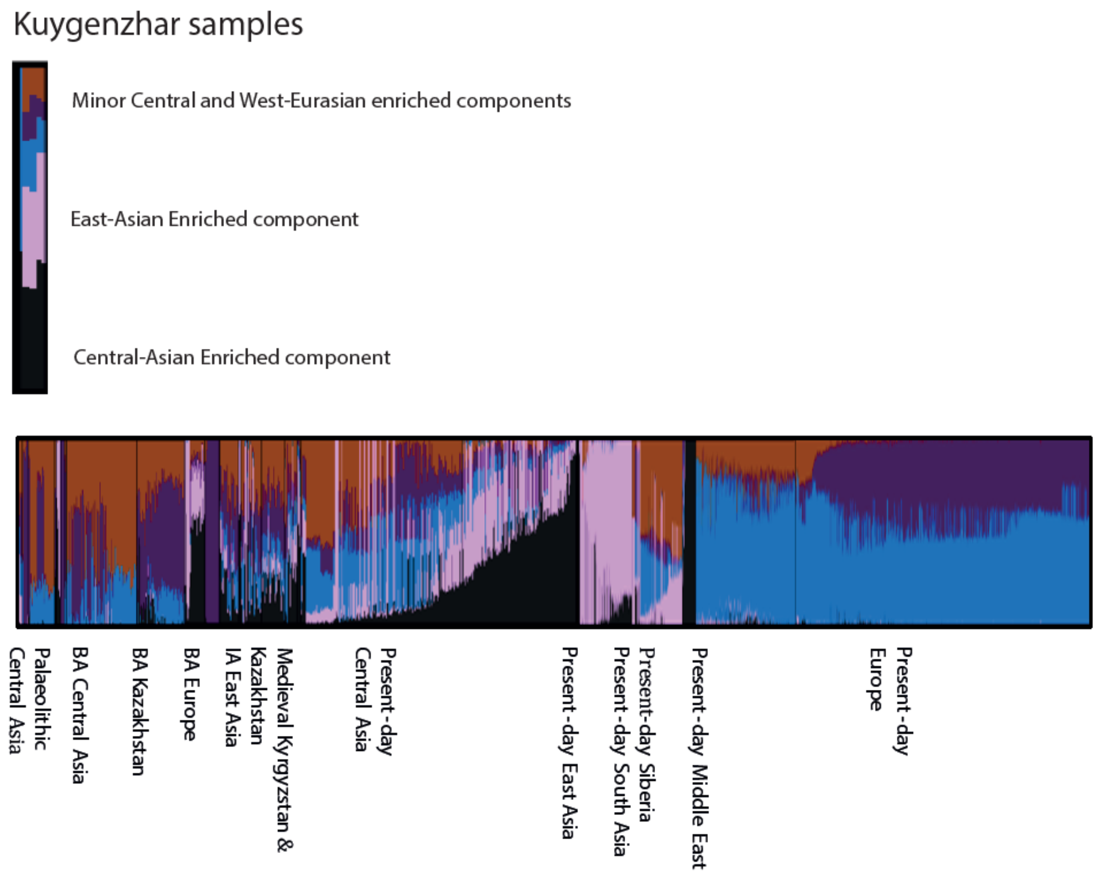

Figure S6. ADMIXTURE inferred clustering of present-day human populations and ancient Asian populations at K=5 where each column is an individual and the colour the cluster to which they are assigned. The Kuygenzhar individuals are enlarged and visualised at the top side of the figure.

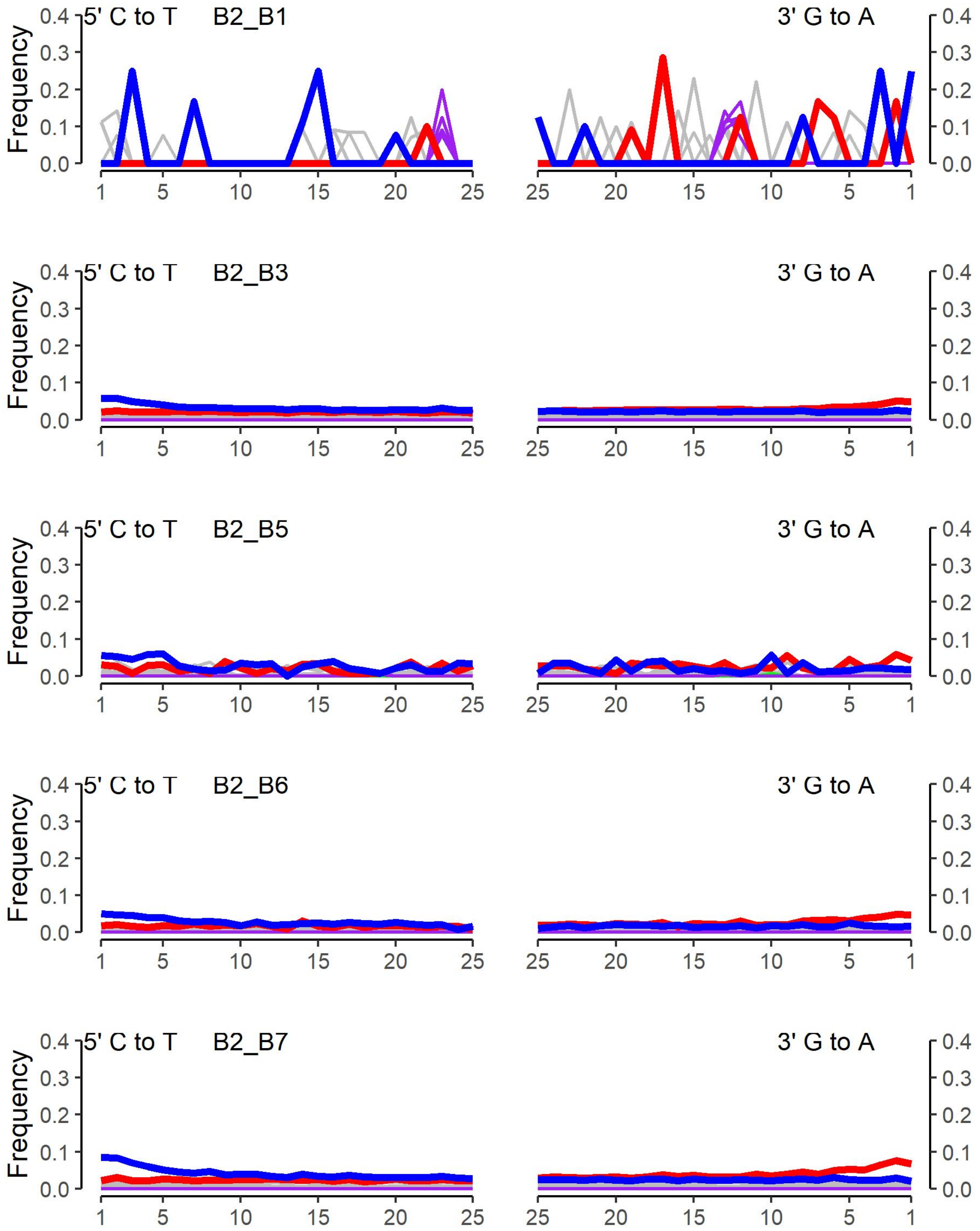

Figure S7. Frequency of aDNA damage patterns at the end of the reads mapped against *T. denticola* reference genome, with special emphasis in substitutions C to T in 5' end (blue) and G to A in 3' end (red). Individuals appear in the following order: B2_B1, B2_B3, B2_B5, B2_B6, B2_B7.

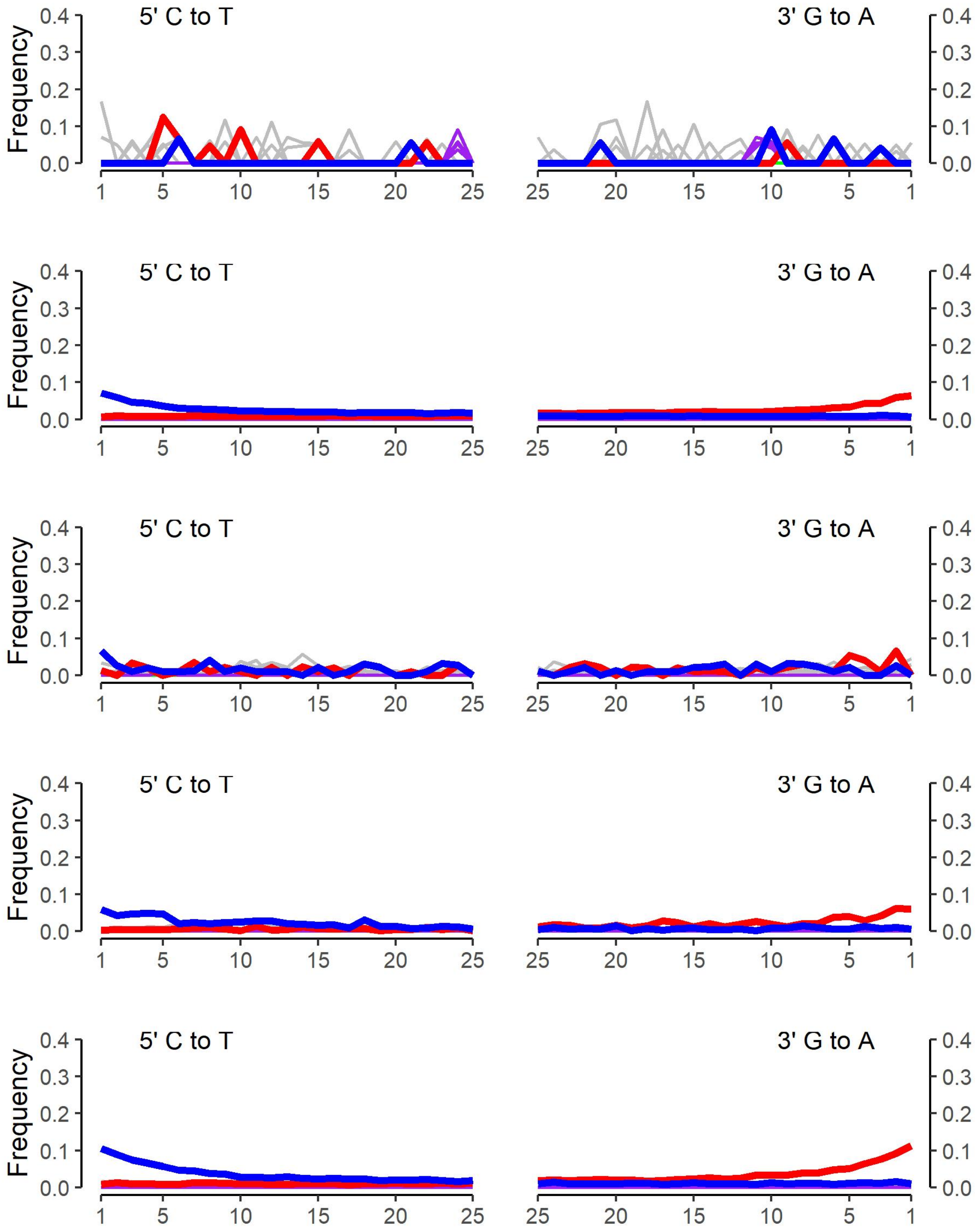

Figure S8. Frequency of aDNA damage patterns at the end of the reads mapped against *P. gingivalis* reference genome, with special emphasis in substitutions C to T in 5' end (blue) and G to A in 3' end (red). Individuals appear in the following order: B2_B1, B2_B3, B2_B5, B2_B6, B2_B7.

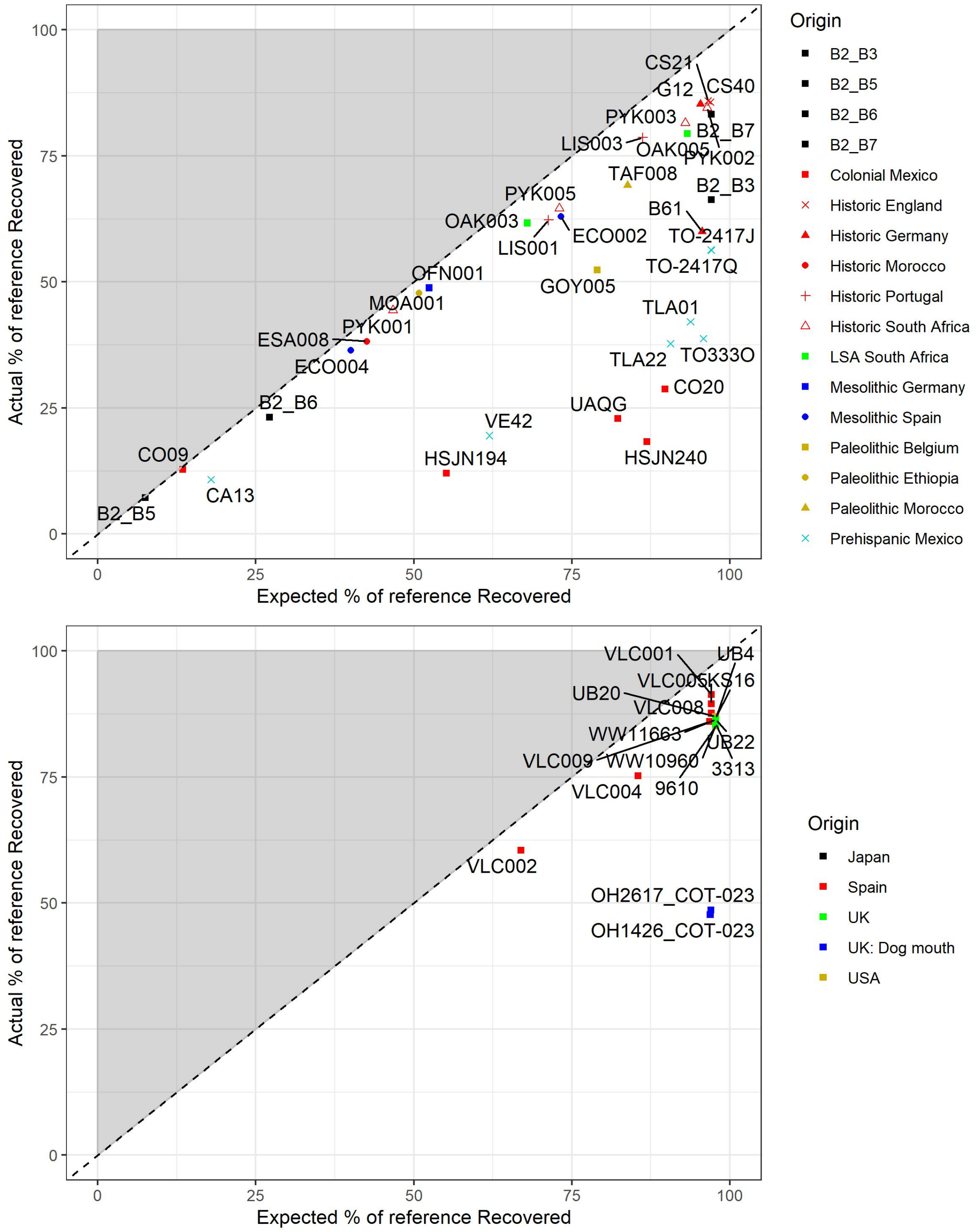

Figure S9. Differences in expected fraction of the reference covered given the length and abundance of reads from different samples. Note that there a cluster of Mexican and European ancient samples that have a retrieved coverage far lower than expected. B2_B3, and dog samples coverage are also lower.

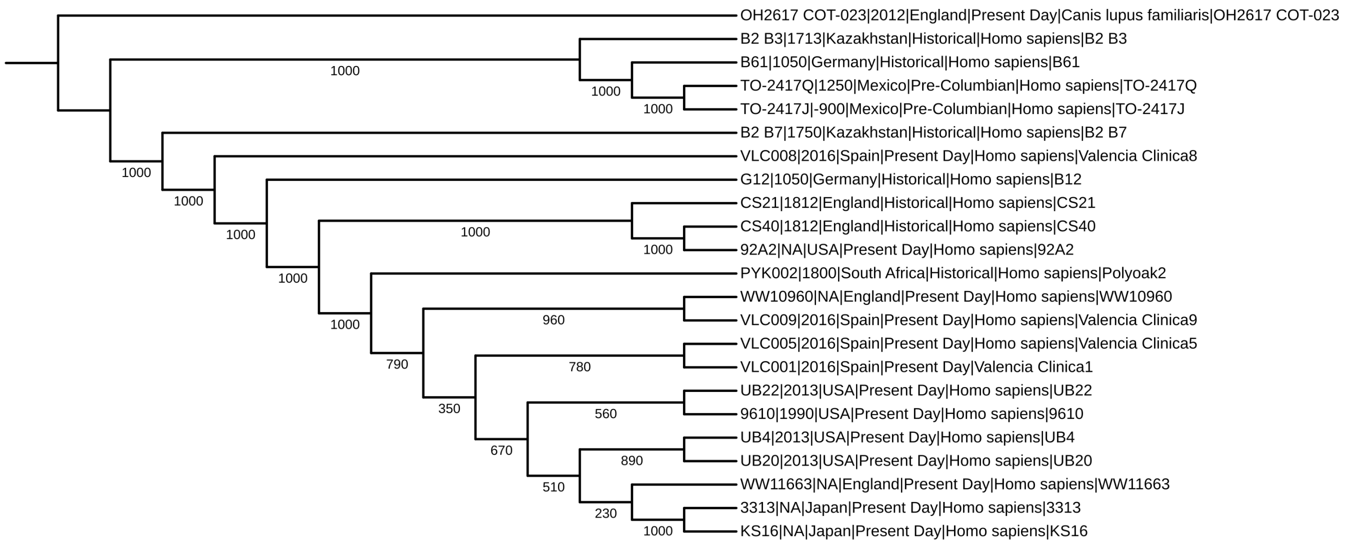

Figure S.10. High quality samples ML tree created with RAxML GTRGAMMA displaying bootstraps (N=1000) for each node. Branch length represented is not real.

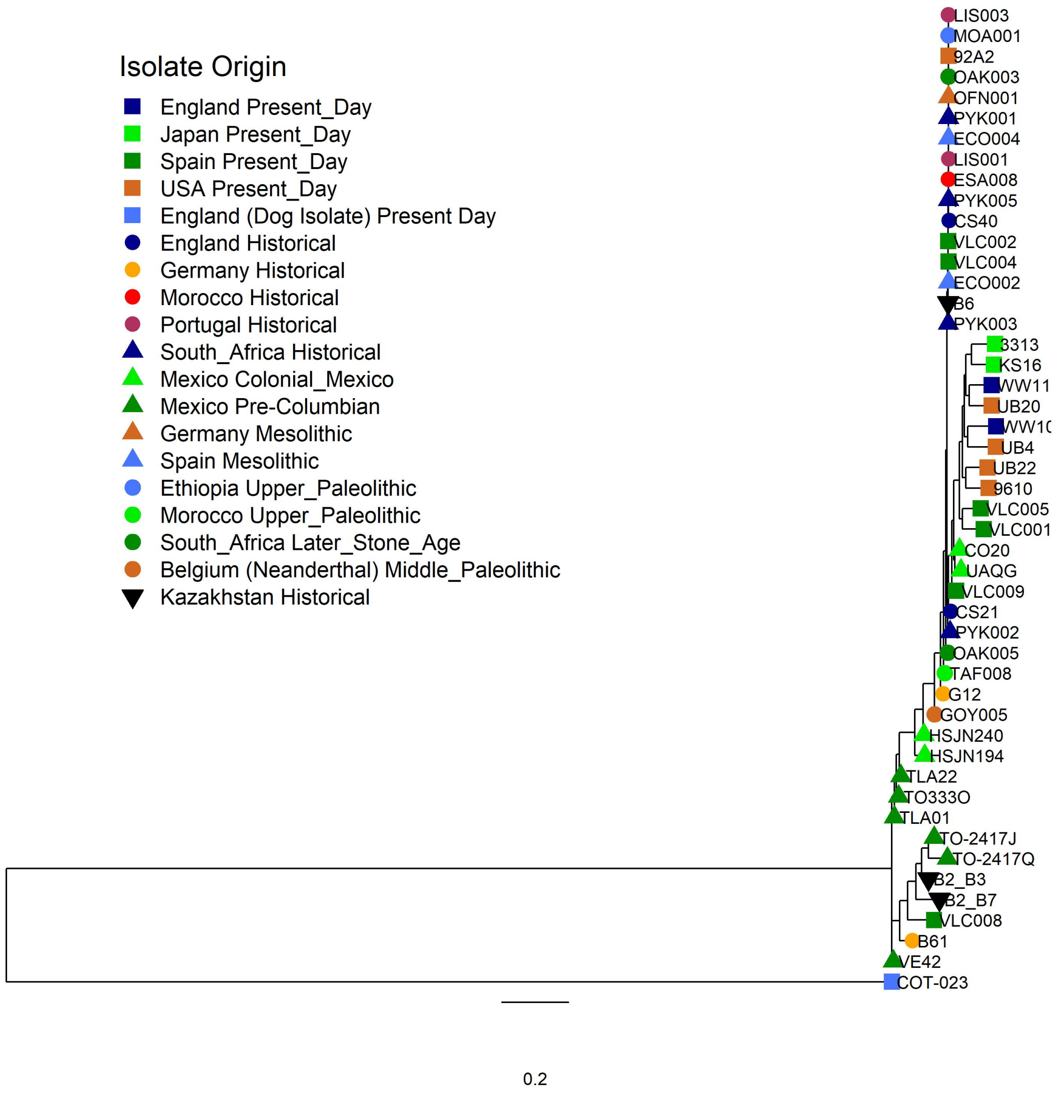

Figure S.11. ML tree created with RAxML GTRGAMMA, using High- and Low-Quality Samples and 100 bootstraps. The tree is rooted to a Dog isolate.

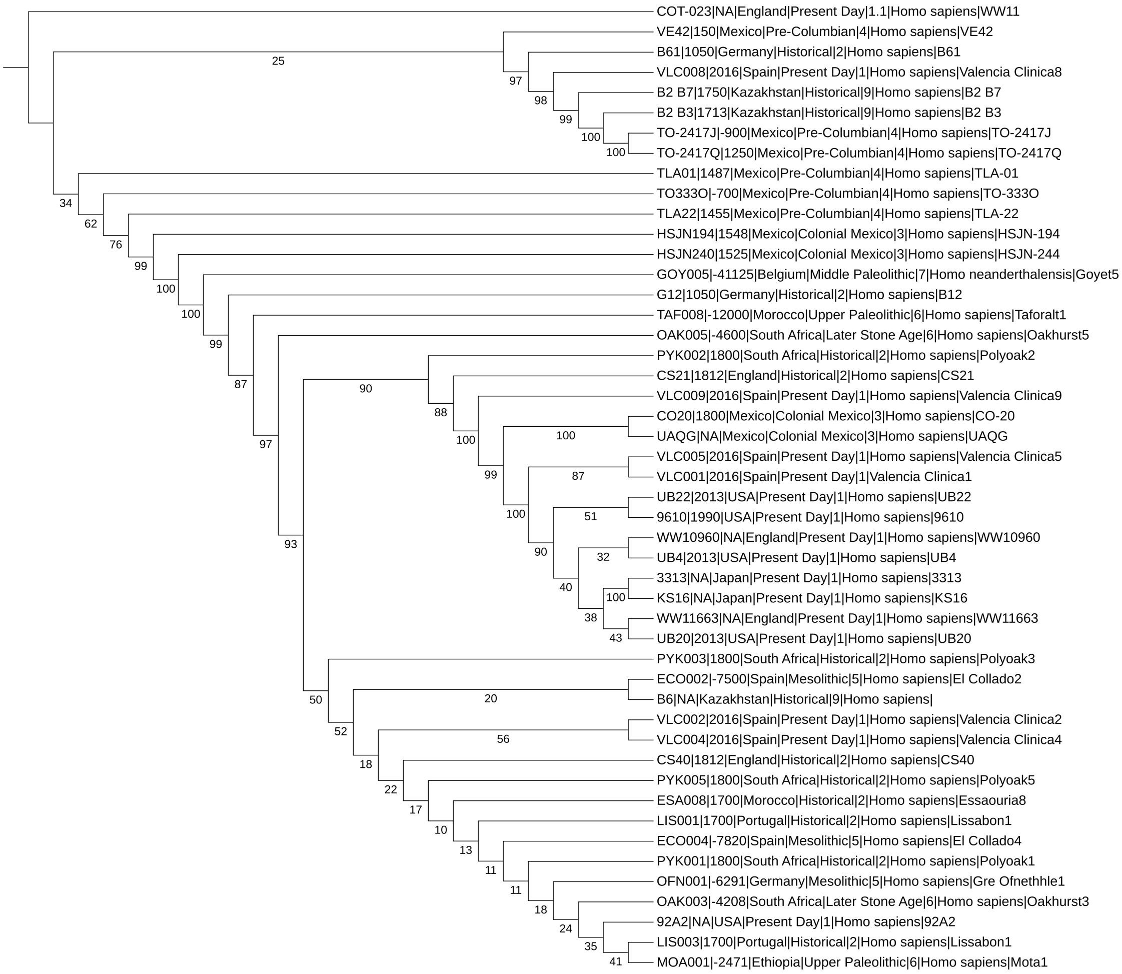

Figure S12. ML tree created with RAxML GTRGAMMA, using High- and Low-Quality Samples and 100 bootstraps. Branch length is ignored. Bootstraps are displayed for each node. The tree is rooted to a Dog isolate.

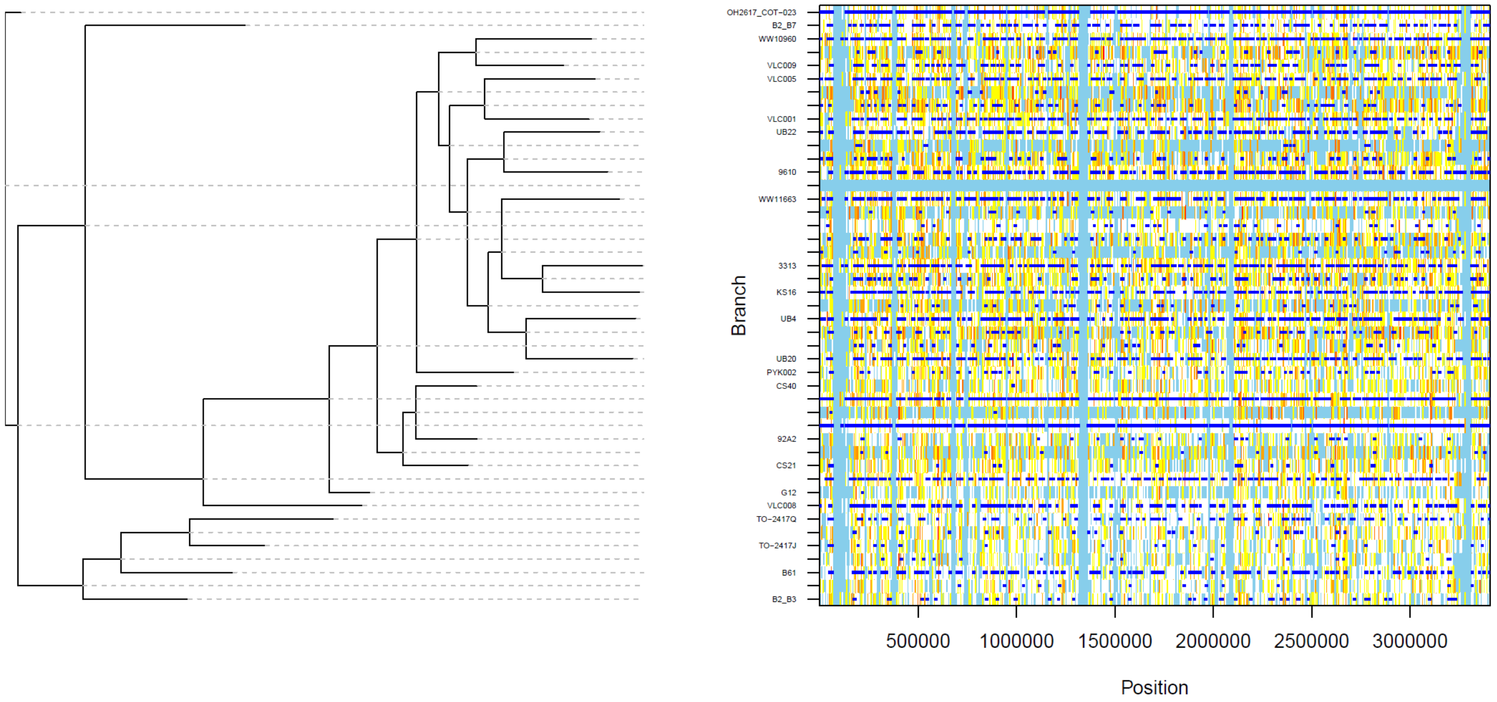

Figure S13. Analysis of recombinations present in the *Tannerella forsythia* alignment with the dog outgroup. Figure provides the ClonalFrameML inferred recombinant tracts with recombination corrected ML tree. The heatmap at right provides a representation of genomic events along the *T. forsythia* chromosome. Recombination tracts are marked as dark blue bars. White bars give non-homoplasic substitutions, while the bars ranging from yellow to red represent homoplasic sites (with a red value denoting a more homoplasic position). Most of the recombinant sites appear within the outgroup and within the node that splits modern diversity and the ancient Mexican clade.

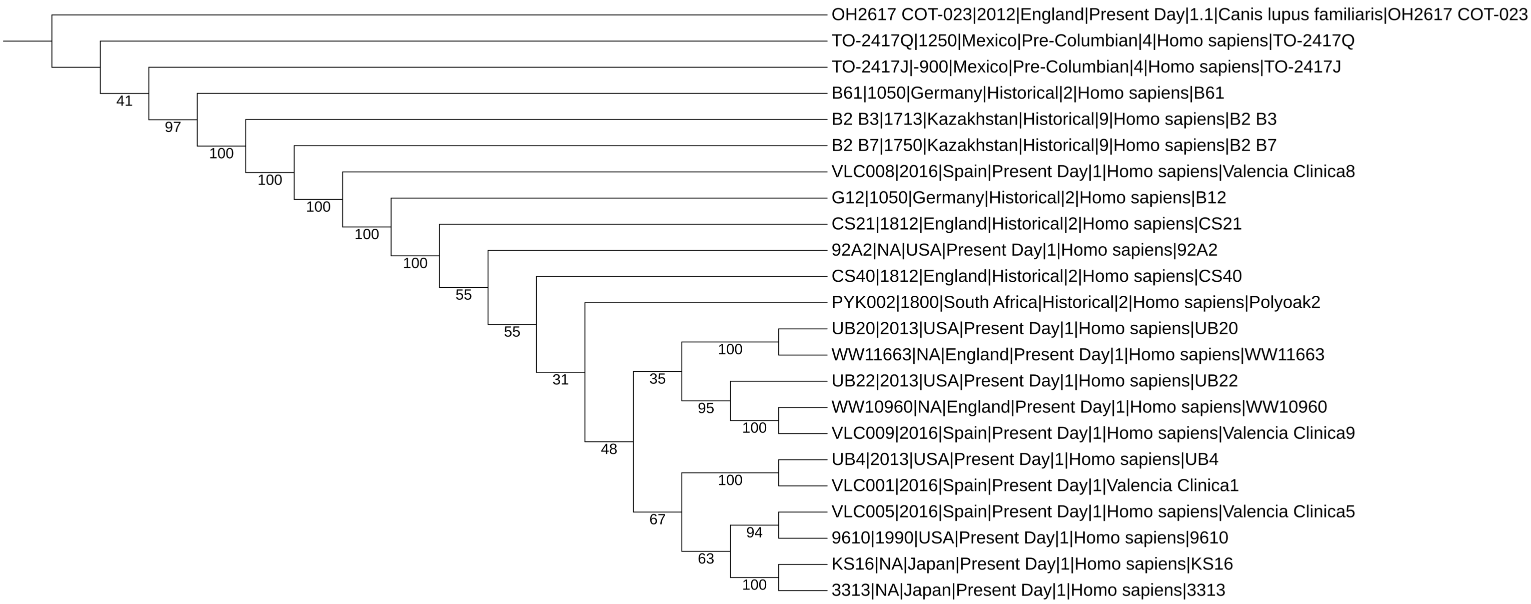

Figure S14. High quality samples ML tree created after recombination and homoplasy pruning. The final alignment used for the tree contained 1494 SNPs. The tree was created with RAxML GTRGAMMA displaying bootstraps for each node (N=100). Branch lengths are set to equal for visual purposes.

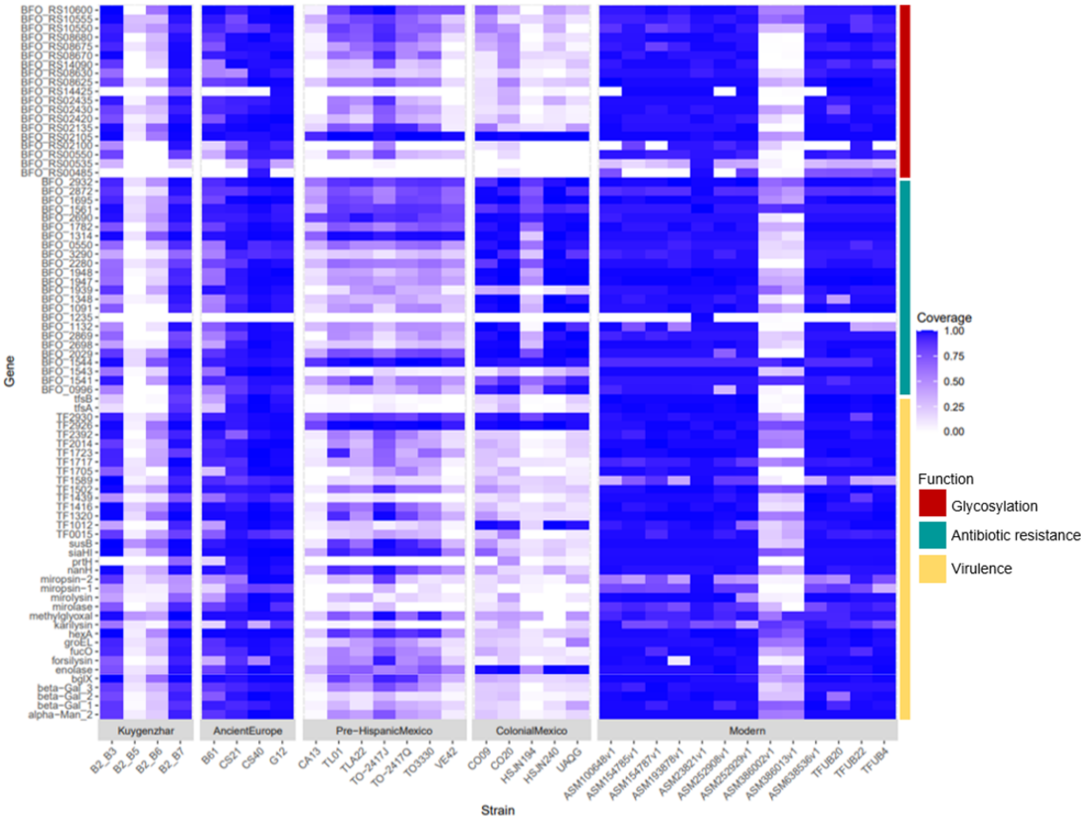

Figure S15: Heatmap displaying the coverage of glycosylation (red), antibiotic resistance (green) and virulence (yellow) genes for ancient Kuygenzhar, European and Mexican strains and modern strains of *Tannerella forsythia*.

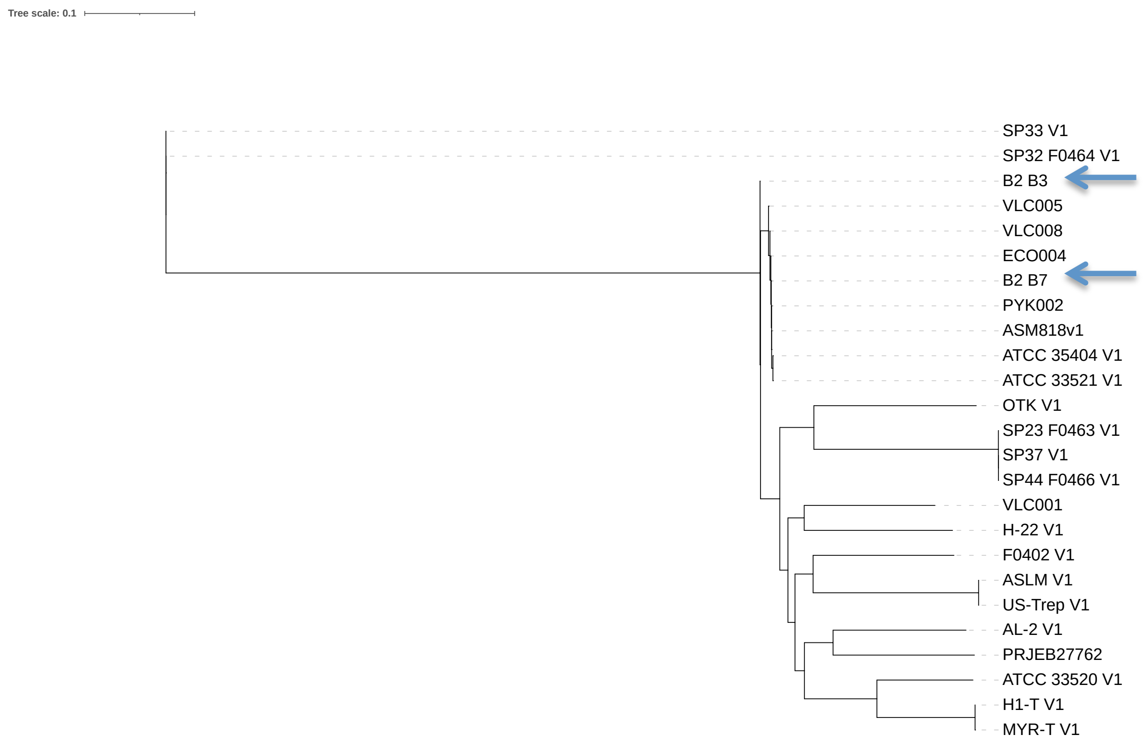

Figure S16: *T. denticola* tree created using 49,963 variant positions. Root was decided based on the NCBI blast-generated dendrogram. Includes samples in this study (B2-B3 and B2-B7) (blue arrows), in Yates et al 2021 (VLC005, VLC008, ECO004 and VLC001), and published assemblies (the rest, their ID are in the tree labels).

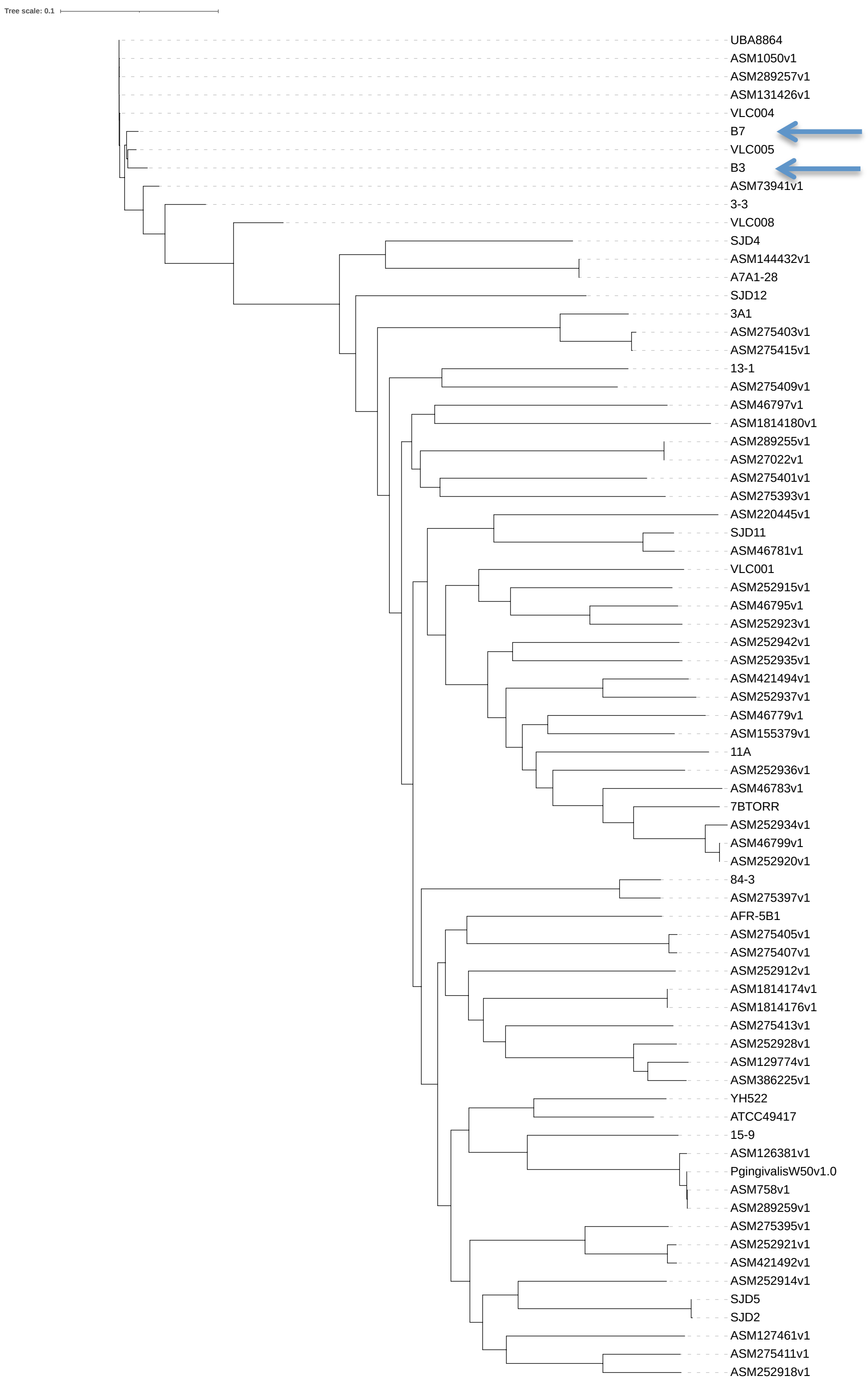

Figure S17: *P. gingivalis* tree created using 88,495 variant positions. Root was decided based on the NCBI blast-generated dendrogram. Includes samples in this study (B2-B3 and B2-B7), in Yates et al 2021 (VLC005, VLC008, VLC004 and VLC001), and published assemblies (the rest, their ID are in the tree labels). Blue arrows mark the Kuygenzhar individuals.
